# Cytotoxicity and Endocrine Disruption in Materials Used for Removable Orthodontic Retainers: A Comprehensive Review

**DOI:** 10.3390/dj13060269

**Published:** 2025-06-17

**Authors:** Katarzyna Chojnacka, Marcin Mikulewicz

**Affiliations:** 1 Department of Advanced Material Technologies, Faculty of Chemistry, Wroclaw University of Science and Technology, 50-372 Wroclaw, Poland; 2 Department of Dentofacial Orthopedics and Orthodontics, Division of Facial Abnormalities, Medical University of Wroclaw, 50-425 Wroclaw, Poland; marcin.mikulewicz@umw.edu.pl

**Keywords:** orthodontic retainers, bisphenol a (bpa), biocompatibility, thermoplastics, polymethyl methacrylate (pmma), clear aligners, microplastic release

## Abstract

**Objective:** To evaluate the cytotoxicity and endocrine-disrupting potential of materials used in removable orthodontic retainers. **Methods:** A literature search (2015–2025) covered in vitro cytotoxicity, estrogenicity, in vivo tissue responses, and clinical biomarkers in PMMA plates, thermoplastic foils, 3D-printed resins, PEEK, and fiber-reinforced composites. **Results:** Thirty-eight in vitro and ten clinical studies met inclusion criteria, identified via a structured literature search of electronic databases (2015–2025). Photopolymer resins demonstrated the highest cytotoxicity, whereas thermoplastics and PMMA exhibited predominantly mild effects, which diminished further following 24 h water storage. Bisphenol-type compound release was reported, but systemic exposure remained below regulatory limits. No statistically significant mucosal alterations or endocrine-related effects were reported in clinical studies. **Conclusions:** Retainer materials are generally biocompatible, though data on long-term endocrine effects are limited. Standardized biocompatibility assessment protocols are necessary to enable comparative evaluation across diverse orthodontic materials. Single-use thermoplastics contribute to microplastic release and pose end-of-life management challenges, raising concerns regarding environmental sustainability.

## 1. Introduction

Removable orthodontic retainers maintain dental alignment after active treatment. Common types include PMMA-based Hawley retainers, composed of a polymethyl methacrylate base and metal wire, and vacuum-formed thermoplastic retainers made of PET-G, polypropylene, or polyurethane [1,2]. Extended intraoral use, especially in relapse-prone malocclusions and hypodontia, raises concerns about material biocompatibility. Studies published between 2015 and 2025 have reported the leaching of bisphenol-A (BPA) and bisphenol-S (BPS), along with cytotoxic effects, including oxidative stress and DNA damage [3,4,5]. Detectable levels of salivary BPA have been observed in individuals wearing both PMMA and thermoplastic retainers [2]. In vitro studies further confirm bisphenol release and associated cellular toxicity across various aligner systems [6,7]. Continuous intraoral exposure to these compounds, even at low concentrations, may contribute to adverse biological effects. This review uniquely focuses on removable orthodontic retainers and integrates clinical biocompatibility, endocrine-disruption, and environmental considerations within a One Health framework.

This systematic review summarizes current evidence on the cytotoxicity and endocrine-disrupting potential of materials used in removable orthodontic retainers, with attention to clinical relevance and environmental implications.

## 2. Materials and Methods

Inclusion and exclusion criteria
Electronic databases including PubMed, Scopus, and Web of Science were searched for relevant studies published between January 2015 and December 2025. The following search terms were used in various combinations with Boolean operators:
“orthodontic retainers” AND “cytotoxicity”;
“removable appliances” AND “endocrine disruption”;
“PMMA” OR “polyurethane” OR “copolyester” AND “toxicology”;
“BPA” OR “BPS” OR “phthalates” AND “release”;
“in vitro” OR “clinical study” AND “orthodontic materials”.
 * *
Studies were included if they met the following criteria:
Peer-reviewed original in vitro, in vivo, or clinical research;
Use of materials employed in removable orthodontic retainers (e.g., PMMA, thermoplastics, polyurethane);
Assessment of cytotoxicity, hormonal activity, or chemical release (e.g., bisphenols, plasticizers);
Human-derived cells, animal models, or human participants used;
Available in English.
 * *
Exclusion criteria:
Studies focused solely on fixed orthodontic appliances or unrelated dental materials;
Review papers, editorials, conference abstracts, or case reports lacking original data;
Articles that did not evaluate biological or toxicological effects;
Studies without full-text access or with insufficient methodological detail.
 * *
No formal protocol was registered (e.g., PROSPERO), but the search strategy was predefined, recorded internally, and consistently applied across all databases.

## 3. Materials Used in Removable Retainers

Removable retainers can be broadly categorized by their material composition: acrylic-based (PMMA) appliances like the Hawley retainer, and thermoplastic appliances like Essix-type clear retainers. Table 1 summarizes the composition of these materials and their known biocompatibility issues. Hawley Retainers (PMMA-based): Hawley retainers consist of a rigid PMMA acrylic plate (typically covering the palate or lingual areas) with embedded metal clasps or wires for retention on teeth. PMMA is formed by polymerizing methyl methacrylate monomers; however, polymerization is rarely 100% complete [3]. A small percentage of residual monomer can remain in the cured acrylic [8]. This residual methyl methacrylate (MMA) is a known irritant and can leach out into saliva, especially in the first days of use. If the acrylic is heat-cured under pressure, conversion is higher (less residual monomer) compared to auto/chemical-cured (cold-cure) acrylic [2]. Apart from PMMA, the Hawley’s metal components (stainless steel wire) can release trace metal ions, which are not addressed in this review. PMMA itself does not contain BPA or typical estrogenic additives—it belongs to a different polymer class—but its residual monomers and additives (e.g., hydroquinone inhibitor, peroxide initiator byproducts) could contribute to cytotoxicity [9]. Acrylic materials modified with bioactive glasses such as Biomin C and S53P4 have also been shown to release beneficial ions like calcium and phosphate in acidic environments, supporting their potential for remineralization applications [10]. Essix Retainers (Thermoplastics): “Essix” is a common term for clear vacuum-formed retainers, introduced by Sheridan in the 1990s, that are fabricated from thin thermoplastic sheets molded to the patient’s teeth [11]. Originally, many Essix retainers were made from PET-G (polyethylene terephthalate glycol-modified), a clear thermoplastic. Other polymer types used include polypropylene and thermoplastic polyurethanes (TPU), as well as multilayer or proprietary blends (for example, Invisalign’s SmartTrack aligners are a type of TPU blend). These materials are typically marketed as medical-grade and BPA-free [4]. However, some polyester-based thermoplastics may use additives for clarity and toughness that are bisphenol derivatives [12]. For instance, PET-G itself is BPA-free, but certain polycarbonate-based or co-polyester variants could contain BPA. Modern orthodontic thermoplastics (Duran^®^, Essix ACE^®^, Zendura^®^ FLX, etc.) have largely eliminated BPA; although materials like SmartTrack are marketed as ‘100% BPA-free’, trace levels of other bisphenol analogs (e.g., BPS) or estrogenic additives may still be present depending on the formulation and manufacturing process. Independent evaluation beyond marketing claims remains essential for accurate biocompatibility assessment [13]. Nonetheless, even “BPA-free” plastics can leach other xenoestrogens (such as bisphenol-S or phthalates) or degradation products. Thermoplastics generally have lower amounts of leachable components than freshly cured PMMA, since they are industrially polymerized, but any residual oligomers, plasticizers, or stabilizers could potentially diffuse out into saliva, especially when the appliance is new or under mechanical stress. 3D-Printed Retainers: an emerging category includes directly 3D-printed orthodontic retainers fabricated from photopolymer resins. These are not yet as common clinically as Essix or Hawley retainers, but they represent a possible alternative material. Three-dimensional printed dental resins often contain methacrylate-based oligomers (some derived from bisphenol-A glycidyl dimethacrylate, etc.). If not properly post-cured and cleaned, they can leach significant monomer. Early evidence suggests some 3D printed retainer materials may have higher cytotoxicity and genotoxicity than thermoplastics [14]. Thus, while promising for custom fabrication, these resins must be carefully evaluated and processed to ensure biocompatibility. Others investigated the leaching behavior of various clear aligner systems and detected the release of multiple chemical compounds, raising concerns about their biocompatibility [7].

## 4. Cytotoxic Effects on Oral Cells

A key indicator of biocompatibility is the potential of retainer materials to induce cytotoxic effects in oral tissues. Patients often wear retainers against the palate or mucosa for many hours a day, so even subtle cytotoxic effects could cause mucosal irritation or cell turnover changes. This issue has been investigated using in vitro cell culture assays, in vivo animal models, and clinical studies measuring biomarkers in retainer-wearing patients. A total of 38 in vitro studies published between 2015 and 2025 were screened based on predefined eligibility criteria. Studies were included if they evaluated cytotoxicity using recognized cell viability assays (e.g., MTT, LDH, live/dead staining) on human oral cell lines or mammalian models, tested materials used in removable orthodontic retainers (e.g., PMMA, polyurethane, PETG, 3D resins), and reported quantitative viability outcomes. Studies focusing solely on fixed appliances, lacking original data, or failing to describe extraction conditions were excluded. The most representative and methodologically transparent studies are summarized in Table 2 to illustrate the range of cytotoxic responses observed across materials.

### 4.1. In Vitro Cytotoxicity Evidence

Multiple in vitro studies have assessed cell viability after exposure to retainer or aligner materials. A common method is the MTT assay on cultured oral cells (gingival fibroblasts or epithelial cells) to detect metabolic activity reduction (a sign of cytotoxicity). Overall, most studies indicate that both PMMA and thermoplastic retainer materials exhibit only slight cytotoxicity in vitro, with cell viability typically above thresholds for biocompatibility [6].

Recent in vitro studies show that orthodontic retainer materials exhibit low to moderate cytotoxicity. In most cases, cell viability remains above 70–90%, even under worst-case extraction conditions [15,16]. Notably, thermoplastic retainer/aligner materials (e.g., PETG- or polyurethane-based) tend to cause only slight reductions in oral cell viability [17]. For example, a comparative study of four popular clear thermoplastic retainer brands (Duran^®^, Biolon^®^, Zendura^®^, and Invisalign^®^ SmartTrack) found all caused only mild cytotoxicity to human gingival fibroblasts (HGFs) in vitro [15]. In vitro comparisons indicate that material composition can influence cytotoxicity—with polycarbonate-based plastics showing higher monomer release than PETG or multilayer polyurethane under laboratory conditions [16,18].

A recent in vitro study also evaluated the cytotoxicity of newly introduced 3D-printed retainer materials compared to conventional thermoplastics. For instance, Al Mortadi et al. [19] tested a proprietary photopolymer resin (Dental LT) and an ethanol-based hard resin (E-Guard) for their effects on cell viability. All materials showed only slight cytotoxicity, and viability increased over time, suggesting that residual leachables dissipate or cellular adaptation occurs [19]. Interestingly, the 3D-printed materials had a slightly higher initial cytotoxic impact than SmartTrack—E-Guard resin reduced cell viability the most on day 1, whereas SmartTrack aligner material exhibited the lowest cytotoxicity among the thermoplastics tested, with cell viability consistently exceeding 90% in human gingival fibroblast assays [15,20,21]. By comparison, Biolon and certain 3D-printed resins exhibited greater reductions in cell viability, depending on post-processing conditions. By day 7, viability in all groups increased significantly, suggesting that proper post-curing and short-term soaking of 3D-printed retainers can mitigate initial cytotoxic leachates [19]. These in vitro findings highlight the potential benefits of thorough post-curing and rinsing of 3D-printed appliances to reduce cytotoxic leachate release [19,21].

Modern materials like polyether-ether-ketone (PEEK), introduced as alternative retainer wires and plates, have shown excellent biocompatibility with minimal cytotoxicity. PEEK is an inert, non-metallic polymer with high strength and proven biocompatibility and stability in biomedical use [22]. Initial clinical applications of PEEK for fixed lingual retainers have shown no adverse tissue reactions. In vitro studies reported low cytotoxicity in fibroblast cultures, consistent with PEEK’s well-documented chemical inertness [23]. This material offers an aesthetic, metal-free retainer option with higher compatibility (e.g., safe for MRI) and minimal cell toxicity or inflammatory response in comparison to conventional stainless-steel wires [23]. By contrast, fiber-reinforced composite (FRC) retainers—another aesthetic alternative—have raised some biocompatibility concerns. Glass-fiber or quartz-fiber reinforced resin bars can leach resin components; indeed, recent studies found that certain FRC retainers significantly reduced oral fibroblast viability, particularly if the resin is exposed or under acidic conditions [24]. Experimental data under acidic conditions indicate that incomplete resin encapsulation or low oral pH may increase monomer release from FRC retainers. In one comparative experiment simulating cariogenic challenges, conventional multistrand wire retainers showed even higher cytotoxicity under low pH (due to metal ion release, e.g., Ni or Cr) than fiber composites—an important reminder that metallic components are not entirely inert either. Corrosion of stainless steel or NiTi retainer wires in an acidic oral environment can release ions (Ni^2+^, Cr^6+^ etc.) that harm cells, potentially explaining the greater cytotoxicity observed with a wire retainer in a low-pH setting [25]. These findings indicate that both polymeric and metallic retainers can exhibit cytotoxicity when degradation products are released, although typically at low levels.

**Table 2:** In vitro studies evaluating the cytotoxic potential of orthodontic retainer materials using cell viability assays (e.g., MTT, live/dead staining) in oral fibroblasts and epithelial cells.

Study (Year)	Retainer Material(s) Tested	Model/Cells	Key Findings on Cytotoxicity	Study Design
Martina et al. [15]	Four thermoplastic brands: Duran (PETG), Biolon (polycarbonate), Zendura (polyurethane), SmartTrack (polyurethane multilayer)	HGF cells; MTT assay	All materials caused only slight cytotoxicity (viability > 80%). Biolon showed the greatest toxicity (most reduction in viability), followed by Zendura and SmartTrack, with Duran showing the least effect (highest viability) Thermoforming the plastics did not eliminate cytotoxic agents and in some cases slightly increased cytotoxicity.	In vitro
Campobasso et al. [25]	- 3D-printed aligners fabricated with Tera Harz TC-85DAC resin (Graphy, Korea) -Post-cured using two different methods: P1: Tera Harz Cure system with nitrogen atmosphere (14 min) P2: Form Cure machine (30 min per side; total 60 min)	MC3T3-E1 mouse pre-osteoblasts Cultured in DMEM; viability measured using the MTT assay at days 7 and 14	P1 group (Tera Harz Cure + nitrogen): Showed no cytotoxicity Cell viability exceeded 100% at both time points (107.1% ± 17.5% on day 7, 106.7% ± 18.4% on day 14) Comparable or even slightly better than control P2 group (Form Cure): Exhibited moderate cytotoxicity Cell viability significantly reduced: 59.8% ± 10.1% on day 7, 47.1% ± 20.6% on day 14 Significantly less biocompatible than P1 and control (*p* < 0.001) Conclusion: Post-curing technique significantly impacts the cytotoxicity of 3D-printed aligners. The P1 method with nitrogen atmosphere is highly biocompatible, while the P2 method may leave residual monomers causing moderate cytotoxicity.	In vitro
Nemec et al. [21]	Invisalign SmartTrack aligner (polyurethane)—inner vs. outer surface (as-grown cells)	Human oral keratinocytes; live/dead staining; PCR	No acute cytotoxicity—very few dead cells observed on aligner surfaces. Cell proliferation was lower on aligner vs. plastic control, indicating a slight growth-inhibitory effect. Notably, aligner-contact cells showed upregulated inflammatory and barrier-function genes. Conclusion: SmartTrack material is non-cytotoxic to oral epithelial cells but can alter cell behavior, inducing a pro-inflammatory gene expression profile.	In vitro
Al Naqbi et al. [26]	Vivera^®^ retainers (Invisalign^®^-associated polyurethane thermoplastic) Tested in two conditions: As-received retainers Used retainers (collected after clinical use)	MCF-7 cells (human breast adenocarcinoma, estrogen receptor-positive—for estrogenicity testing) MDA-MB-231 cells (estrogen receptor-negative control) NIH/3T3 mouse fibroblasts (for general cytotoxicity)	No cytotoxic effect was found on fibroblasts for eluates from either as-received or used retainers. No estrogen receptor-mediated proliferation was observed in MCF-7 cells exposed to either retainer sample. No proliferative effect on estrogen-insensitive MDA-MB-231 cells. Authors conclude that Vivera^®^ retainers do not exhibit acute cytotoxicity or estrogenicity under the tested conditions. The study supports good short-term biocompatibility of the retainer material.	In vitro

In summary, the last decade of research affirms that current orthodontic retainer materials are largely biocompatible, causing only mild cytotoxic effects on oral cells in vitro and minor, transient cellular stress in vivo [5,6]. Still, detectable differences exist between material types and brands. New materials like PEEK show promise in further minimizing cytotoxicity due to their chemical stability and inertness [23].

### 4.2. In Vivo and Clinical Evidence of Cytotoxic Effects

Encouragingly, in vivo studies in humans over the past decade have not revealed any severe cytotoxic reactions to retainer materials, but they do corroborate some subtle biological effects. A groundbreaking randomized controlled trial monitored patients wearing either a Hawley acrylic retainer or an Essix clear retainer for signs of cellular damage. Salivary biomarkers of DNA damage (8-hydroxy-2′-deoxyguanosine, 8-OHdG) and antioxidant response (Nrf2, Keap1) were measured, alongside cytologic examination of patients’ buccal mucosa cells for nuclear abnormalities. The Hawley retainer group showed a significant rise in 8-OHdG in saliva after 1 and 3 months, indicating increased oxidative DNA damage likely due to leached monomer (residual methyl methacrylate or additives from the acrylic). In contrast, the Essix (vacuum-formed thermoplastic) group did not have elevated 8-OHdG; intriguingly, their levels slightly decreased over time. This suggests the chemically cured acrylic retainer induced more oxidative stress than the thermoplastic. However, mucosal cell exams told a complementary story: patients in the Essix group had a higher frequency of micronuclei and other nuclear anomalies in cheek cells after 2–3 weeks than those in the Hawley group. Both types of appliances were associated with an increase in cellular turnover and some degree of cell nuclear damage (vs. pre-treatment baseline), but Essix retainers led to more epithelial cell micronuclei, whereas Hawley retainers led to more oxidative DNA damage in saliva. These clinical findings align with the notion that each material has distinct interactions with tissues: the acrylic may leach small amounts of peroxide or monomer causing systemic oxidative stress, while the plastic may cause more direct mechanical or frictional stress on the mucosa (hence micronuclei) [5].

## 5. Estrogenic Potential and BPA Release

Perhaps the most publicized biocompatibility concern with dental plastics is the possibility of endocrine disruption via leaching of estrogen-mimicking chemicals. The prime example is Bisphenol-A (BPA), a known xenoestrogen used in polycarbonate plastics and epoxy resins. BPA can bind estrogen receptors (albeit with lower affinity than natural estradiol) and has demonstrated endocrine-disrupting effects, including developmental and reproductive toxicity [27]. Orthodontic retainers and aligners, being plastic products in the mouth, have raised concerns about the potential release of BPA or structurally related xenoestrogens into saliva. The clinical relevance of any potential release remains uncertain, particularly with regard to systemic estrogenic effects.

### 5.1. BPA Release from Retainers: In Vitro vs. In Vivo

Early in vitro studies generally detected no measurable BPA release or reported concentrations below analytical detection thresholds (e.g., <1 ng/mL) from clear aligners under laboratory conditions. For instance, Schuster et al. [28] and Gracco et al. [29] detected no BPA or notable monomer release from Invisalign aligners soaked in artificial saliva. Katras et al. [30] recently tested several brands (SmileDirectClub, Invisalign, Essix ACE) in various media (saliva, gastric fluid, ethanol) and found that any BPA released occurred mostly in the first 24 h, and the levels were below established safety thresholds. These controlled tests often used high-performance liquid chromatography (HPLC) or mass spectrometry to look for BPA in solution; results were often at or below detection limits for new aligners [6]. BPA release levels and estrogenic effects of orthodontic retainer materials, based on in vitro studies are discussed in Table 3.

In vitro estrogenicity assays have been employed to test whether retainer leachates can stimulate estrogen-sensitive cells. Two independent studies [18,26] used the MCF-7 breast cancer cell proliferation assay—an indicator of estrogen receptor activation—to evaluate aligner and retainer materials. Both found no estrogenic effect: exposure to Invisalign^®^ aligner material or Vivera^®^ retainer material did not induce proliferation of estrogen-sensitive MCF-7 cells above controls. In these assays, positive controls (17β-estradiol or BPA) induced marked proliferation of MCF-7 cells, whereas aligner samples exhibited proliferation rates comparable to negative controls. Similarly, no effect was seen on estrogen-insensitive cell lines (MDA-MB-231), confirming that the retainer materials themselves lacked estrogen-receptor-mediated activity. These results align with chemical analyses showing that BPA release from today’s orthodontic polymers is minimal. Advanced analytical techniques, such as gas chromatography-mass spectrometry (GC-MS) and liquid chromatography-tandem mass spectrometry (LC-MS/MS), have been used to detect bisphenol traces in aligner material extractions [18,26]. In several studies between 2016 and 2021, researchers could not detect any BPA or related monomers in saliva or artificial saliva immersions of new clear aligners over extended periods [6].

However, clinical studies have told a more cautionary tale. Raghavan et al. [2] conducted a randomized clinical trial measuring salivary BPA in patients wearing different retainers. They randomized 45 patients to one of three groups: (1) Essix vacuum-formed retainer, (2) Hawley retainer (heat-cured PMMA), and (3) Hawley retainer (cold-cure/autopolymerized PMMA). Saliva samples were collected before retainer insertion, then at 1 h, 1 week, and 1 month after. All groups exhibited a significant increase in salivary BPA following retainer placement (*p* ≤ 0.05) [2].

A follow-up randomized trial by Nanjannavar et al. [12] tested a simple intervention: pre-soaking the retainer in water for 24 h before giving it to the patient. Remarkably, by immersing the vacuum-formed retainers in 37 °C water overnight, the salivary BPA levels in patients were dramatically reduced: at 1 h, pre-soaked retainers yielded only ~0.07 ppm BPA versus 0.33 ppm in unsoaked retainers. At 1 week and 3 weeks, the pre-soaked group’s BPA was near zero. These findings suggest that most leachable BPA can be eliminated through a 24 h water soak prior to clinical use, providing a practical approach to reduce patient exposure [12].

It is worth noting that scientific understanding of BPA’s safety threshold has evolved. Earlier, agencies like the U.S. FDA and EPA set relatively high “tolerable daily intake” values (~50 µg/kg body weight/day). However, more recent evidence suggests that adverse endocrine effects might occur at much lower doses than previously thought. Animal and epidemiological studies have linked chronic low-level BPA exposure to subtle hormonal disturbances, prompting regulatory bodies (especially in the EU) to drastically lower acceptable exposure limits in 2021–2023. For example, the European Food Safety Authority recommended reducing the safe daily BPA intake by orders of magnitude (down to nanogram/kg levels) due to uncertainties about immune and developmental effects at low doses [31]. In parallel, studies have reported that even low-dose BPA exposure can alter immune function in human cells, reinforcing concerns about potential endocrine disruption [32]. In this context, even the very small BPA amounts from orthodontic retainers are being re-evaluated from a precautionary standpoint. To date, no hormonal disorders or systemic endocrine effects have been directly linked to BPA leaching from orthodontic appliances. A review by Hassan et al. [33] highlights the ongoing development of BPA-free adhesives, aligner materials, and smart polymers that combine safety with advanced mechanical and antimicrobial properties, reinforcing the industry trend toward eliminating endocrine-disrupting compounds altogether.

**Table 3:** BPA release levels and estrogenic effects of orthodontic retainer materials, based on in vitro studies, chemical analyses (e.g., HPLC, LC-MS/MS), and clinical trials.

Study (Year)	Materials and Conditions	BPA Release Findings	Estrogenic Effect	Study Design
Katras et al., 2021 [30]	SmileDirectClub, Invisalign, Essix ACE aligners; incubated in artificial saliva, gastric fluid, and 20% ethanol; sampled at 0, 1, 2, 6, 10, 20 days	Low BPA release observed from all aligners, mostly within first 24 h (initial “burst” release). BPA levels remained below 5 µg/L in saliva and below EU safety thresholds at all times. No significant BPA difference between the three brands or between saliva vs. gastric media.	No direct estrogenicity test, but given BPA levels were far below toxicological concern, endocrine effects deemed unlikely. Authors note these BPA amounts are “below established safety levels for adult patients”.	In vitro
Intissar et al., 2020 [34]	Invisalign^®^ aligners (polyurethane); new vs. 2 weeks used; stored in artificial saliva up to 8 weeks	No BPA detected in any aligner extract (HPLC analysis) at <5 ppb detection limit, even after 2 weeks intraoral use and continued saliva storage. Aligners appeared chemically stable with respect to BPA over 8 weeks.	Not applicable (chemical analysis only). Supports that properly cured aligner polymers do not leach BPA in detectable amounts, hence no estrogenic stimulus expected.	In vitro
Raghavan et al., 2017 [2]	Patients (n = 45) wearing: (1) vacuum-formed Essix retainer (PETG), (2) heat-cured acrylic Hawley, (3) chemically cured acrylic Hawley; salivary BPA measured before and 1 month after retainer delivery	Salivary BPA increased in all groups after 1 month of retainer use, but levels differed by retainer type. Chemically cured Hawley: highest BPA (~6–8 µg/L increase on average). Vacuum-formed Essix: moderate increase (~2–3 µg/L). Heat-cured Hawley: lowest increase (~1 µg/L or less). All values were low (parts-per-billion).	No clinical symptoms of endocrine disruption in any group. The BPA levels, while detectable, were below doses known to cause hormonal effects in humans. Authors suggest using heat-cured acrylic or BPA-free materials to minimize exposure.	In vitro
Iliadi et al., 2017 [35]	Experimental BPA-free orthodontic adhesive vs. conventional Bis-GMA adhesive; no direct BPA, uses alternative monomer (phenyl-propanediol dimethacrylate) for bonding fixed retainers	No BPA release by design (formulation contains no BPA or bisphenol derivatives). Compared to a conventional adhesive that can release trace BPA (from Bis-DMA degradation), the experimental adhesive showed undetectable BPA in eluates.	No estrogenic components present; the BPA-free adhesive showed no estrogenic or cytotoxic effects in vitro. It achieved similar bond strength to controls, suggesting viable clinical use. This trend highlights efforts to eliminate BPA sources in orthodontic materials and reduce endocrine-related risks.	In vitro
Eliades et al., 2009 [18]	Three sets of Invisalign aligners immersed in normal saline at 37 °C for 2 months; eluents tested at 5%, 10%, and 20% concentrations	Three sets of Invisalign aligners immersed in normal saline at 37 °C for 2 months; eluents tested at 5%, 10%, and 20% concentrations.	Three sets of Invisalign aligners immersed in normal saline at 37 °C for 2 months; eluents tested at 5%, 10%, and 20% concentrations.	In vitro

In summary, thermoplastic retainers can indeed release BPA into saliva, especially initially, although simple measures (like pre-soaking or choosing alternative materials) can greatly reduce this exposure. Heat-cured acrylics release the least BPA (since they contain none, aside from any external contamination), whereas some thermoplastics have shown transient BPA leaching. Manufacturers have responded by advertising BPA-free aligner materials, but clinicians should remain aware of potential trace chemicals.

### 5.2. Estrogenic Effects of Leached Substances

While BPA detection is straightforward, establishing a definitive estrogenic biological effect remains more complex. Researchers have used estrogen-sensitive cell assays to see if retainer materials can activate estrogen receptors. Results suggest that any BPA or similar chemicals leached were below the threshold to elicit an estrogenic response in vitro [6]. However, endocrine disruption can occur at very low doses in vivo, potentially with non-linear dose–response. There is concern that chronic exposure, even to “low” BPA levels, might have subtle developmental or hormonal effects. No clinical studies have directly linked orthodontic retainer use to hormonal changes or systemic endocrine outcomes (such studies would be difficult to conduct and control). We do know from other dental materials (like sealants and composites) that transient BPA spikes in saliva/urine happen after placement, but these return to baseline in 24–48 h and are considered too low to cause harm [27]. The American Dental Association notes that “trace amounts of BPA may leach out of freshly polymerized resins and cause a small, transient increase in BPA levels in saliva and urine” [27], which aligns with what we see in retainers (initial release then tapering off). An additional angle is whether other xenoestrogens aside from BPA are present. Some plastics might contain bisphenol S (BPS) or phthalate plasticizers, which also can have estrogen-like or anti-androgen effects. Most orthodontic appliance materials are now phthalate-free (DEHP was used in some plastics historically but is not typically in modern aligners). BPS has been used to replace BPA in “BPA-free” plastics, but its safety is also debated. No specific studies have reported on BPS leach from retainers yet. While BPA has been systematically evaluated in multiple studies, references to BPS and phthalates in the context of retainers are based on a limited number of reports and were included to highlight potential but insufficiently studied alternatives.

In summary, current evidence indicates no overt estrogenic effects from Hawley or Essix retainers under standard conditions of use. Measurable BPA can be released, especially from some thermoplastics, but usually at levels considered low. Both in vitro estrogen assays (Yazdi et al. [6]) and clinical observations have so far been reassuring in terms of hormonal impact. Nevertheless, given the widespread use of these appliances, cumulative exposure in adolescent patients (especially for those who might wear aligners for years, then retainers) is an area worthy of continued surveillance. It is also prudent to take simple precautions, such as initially rinsing or soaking new plastic appliances and using BPA-free materials whenever possible, to further minimize any endocrine-disrupting risk [12].

### 5.3. Molecular Mechanisms of Cellular Damage and Estrogen Action

#### 5.3.1. Oxidative Stress and DNA Damage

Residual monomers such as MMA can leach from acrylic retainers, penetrate oral tissues, and enter saliva and systemic circulation, albeit in small quantities [36]. These monomers can undergo metabolic activation or redox reactions that generate ROS (reactive oxygen species). ROS in the oral environment (or within mucosal cells) can oxidize DNA bases, lipids, and proteins, leading to oxidative damage [37]. The increase in salivary 8-OHdG in Hawley retainer wearers [8] is a direct indicator of DNA oxidative injury—8-OHdG is formed when guanine in DNA is oxidized. Normally, the body can repair such damage, but a sustained increase suggests a chronic ROS presence. Notably, in the RCT, Essix wearers did not exhibit elevated 8-OHdG levels—possibly due to lower ROS generation or effective adaptive cellular responses.

The body’s defensive response to oxidative stress often involves the Nrf2/Keap1 pathway. Nrf2 is a transcription factor that, when activated by oxidative signals, moves to the nucleus and upregulates antioxidant genes. Gunel et al. measured Nrf2 and Keap1 levels in their trial as well. They did not find significant differences between Hawley vs. Essix groups in Nrf2 or Keap1 [5], which could imply the oxidative challenge was not strong enough or sustained enough to trigger differential activation of that pathway, or that both groups had similar antioxidant responses.

In addition to oxidative DNA damage, direct cytotoxic mechanisms include disruption of membrane integrity. Acrylic monomer is a small organic solvent that can alter cell membrane integrity. Cell culture studies show that high concentrations of MMA or certain aligner additives can lead to loss of membrane potential and cell lysis. As noted, saliva can mitigate some of this by diluting and binding these monomers [6]. Nonetheless, genotoxicity at the cellular level—such as increased micronuclei—has been observed in patients [5]. Micronuclei are formed when chromosome fragments or entire chromosomes are excluded from daughter nuclei during mitosis—typically as a consequence of DNA strand breaks or mitotic spindle disruption induced by chemical exposure. The elevated frequency of micronuclei observed after 2–3 weeks of Essix retainer use suggests an acute genotoxic response, potentially attributable to initial chemical leachate exposure or mucosal friction [8].

In summary, cytotoxic effects of retainer materials are likely mediated by chemical leachates inducing oxidative stress and direct cell damage. PMMA’s leached MMA is a prime suspect for ROS generation (leading to 8-OHdG formation). Thermoplastics, which have less obvious monomer release, might still shed other agents or even microscopic particles that stress cells. We should also consider mechanical forces: an Essix retainer pressing on the mucosa could cause mild ischemia or pressure that leads to cell turnover and inflammation, indirectly causing oxidative stress. Despite the multifactorial nature of these mechanisms, the presence of oxidative DNA damage markers and indicators of cellular injury points to a measurable, though limited, biological response.

#### 5.3.2. Estrogen Receptor Activation Pathways

If chemicals like BPA leach out, they can bind to estrogen receptors (ERα and ERβ) found in various tissues. In the context of oral tissues, periodontal ligament fibroblasts and bone cells have ERs, but oral epithelium is not a classic estrogen target. However, ingested BPA could act on distant targets (e.g., endocrine organs). BPA binding to ER can mimic estrogen, altering gene transcription. Some effects of chronic low-dose BPA exposure noted in toxicology studies include changes in reproductive organ development, metabolic changes, and behavior alterations [27]. But these are typically from continuous exposure orders of magnitude higher than what a retainer would contribute.

One potential local consequence is that estrogen signaling may influence wound healing and inflammatory responses. If BPA from a retainer is absorbed through oral mucosa, could it affect gingival inflammation or alveolar bone? There is no direct evidence of that to date. While in vitro studies suggest that BPA may modulate inflammatory pathways, no clinical data currently support a direct association between BPA exposure from orthodontic appliances and gingival inflammation. A study on rats or cells would be needed to see if salivary BPA at these levels has any local ER-mediated effect (none has been clearly documented). The lack of MCF-7 cell response to aligner extracts [6] is somewhat reassuring that the effective estrogenic activity of leachates is extremely low. Additionally, BPA has a short half-life in humans (excreted within hours once absorbed). Thus, the transient increase in systemic BPA concentration following appliance insertion is unlikely to sustain estrogen receptor activation over time. Estrogenic effects can exhibit non-monotonic dose–response relationships, wherein lower doses may elicit disproportionately greater responses than moderate exposures due to regulatory feedback mechanisms [27].

In sum, while orthodontic retainer materials can release chemicals capable of binding estrogen receptors, the current data suggest the levels are too low to cause meaningful estrogenic activation in vivo. The molecular pathway is well-understood (xenoestrogen → ER activation → gene expression changes), but fortunately, in the case of modern retainer materials, this pathway appears to be minimally triggered. Nonetheless, prudent material refinement—such as avoiding Bis-DMA monomers that degrade to BPA [27]—remains essential to minimize potential estrogenic activity, and for ongoing research, particularly on any long-term systemic effects in vulnerable populations (young patients, those who wear appliances for many years).

### 5.4. Clinical Significance of Findings for Long-Term Retainer Use

From a clinical standpoint, the central question is whether cytotoxic and estrogenic effects translate into measurable health consequences for patients. Orthodontic retainers are often worn nightly for many years (sometimes lifelong for relapse prevention). In cases such as hypodontia, where permanent solutions are delayed, retainers with prosthetic teeth may be worn daily until adulthood. Thus, understanding the clinical significance of chronic exposure to retainer materials is important.

Mucosal Health and Symptoms: The majority of patients tolerate both Hawley and Essix retainers well, with no overt tissue damage. Nevertheless, case reports and surveys have documented various mucosal reactions. Common issues include:

- Initial irritation: Patients may experience gum or palatal soreness when a new retainer is inserted. Symptoms typically subside as tissues adapt or as residual monomers are eliminated. In the case of aligners/retainers, patients have reported a transient oral discomfort or altered taste perception during the initial period of use, potentially related to chemicals releasing initially [30].
- Ulceration or contact dermatitis: A small subset of individuals can have an allergic contact reaction to PMMA or to something in the plastic. This could manifest as localized redness, ulcers, or even diffuse symptoms like lip swelling and itching. Acrylic allergy is well-documented in dentistry, particularly among denture wearers sensitive to residual MMA. For those individuals, a switch to a different material (e.g., a metal retainer or hypoallergenic lining) may be needed [38].
- Taste disturbance and dry mouth: Some patients initially note a plastic or chemical taste from their retainer. Dry mouth (xerostomia) has also been reported [6], though it remains unclear whether it stems from the appliance’s physical presence or chemical composition. Dry mouth can exacerbate any cytotoxic effect because saliva flow helps buffer irritants.
- Periodontal impacts: Poorly fitting or unclean retainers can cause gingival inflammation. While not directly a chemical toxicity issue, if a retainer causes chronic inflammation, that in itself leads to oxidative stress in tissues. Essix retainers, typically worn only at night after the initial period, have generally been associated with better periodontal outcomes than fixed retainers, primarily due to their removability and ease of cleaning [39]. Any material-related risks are likely offset by proper hygiene practices.

An overview of removable orthodontic retainers and their associated biological risks is provided in Table 4.

## 6. Regulatory Standards and Global Guidelines

Assessment of the cytotoxic and endocrine-disrupting properties of dental materials entails both scientific scrutiny and regulatory oversight. Both the United States Food and Drug Administration (FDA) and the European Union (EU) have established frameworks to ensure the safety of orthodontic appliances, including removable retainers, prior to and during clinical use.

**FDA** **(United States)**

Under FDA classification, removable orthodontic retainers are categorized as Class II medical devices and require 510(k) premarket notification that demonstrate substantial equivalence to existing legally marketed devices [40]. To meet safety requirements, manufacturers must conduct biocompatibility assessments in accordance with ISO 10993 standards [41], which govern biological evaluation of medical devices. These include ISO 10993-5 for cytotoxicity, ISO 10993-10 for irritation and sensitization, and other relevant tests depending on the material’s intended contact duration and anatomical site [41].

For devices intended for long-term mucosal contact, such as retainers, compliance with ISO 10993 protocols is essential to confirm absence of acute or chronic toxicity, genotoxicity, and tissue irritation. Materials such as Invisalign’s SmartTrack have reportedly passed the full ISO biocompatibility suite for mucosal applications, as disclosed by the manufacturer.

With regard to chemical leachates, the FDA has not imposed specific limits on bisphenol A (BPA) content in dental devices. Unlike baby bottles, from which BPA has been banned since 2012, dental appliances are regulated under a risk-based framework. To date, neither the FDA nor the American Dental Association has issued recommendations discouraging the use of BPA-containing dental materials [42]. Some polymers may contain BPA as a trace contaminant or degradation byproduct, but exposure levels from dental sources are typically low and transient [43].

The FDA currently encourages voluntary minimization of BPA exposure, particularly in vulnerable populations, including infants. However, in the absence of evidence indicating adverse outcomes from dental applications, no regulatory prohibition is enforced. As a result, manufacturers have largely adopted BPA-free formulations in response to market expectations rather than regulatory mandate.

**EU** **Regulations**

The European regulatory framework imposes comparatively stricter controls on hazardous substances used in medical devices. Regulation (EU) 2017/745 on medical devices (MDR), fully effective since 2021, mandates that manufacturers evaluate and disclose the presence of carcinogenic, mutagenic, or reprotoxic (CMR) compounds and endocrine-disrupting chemicals. If any component of a device intended for patient contact contains >0.1% by weight of such substances—classified as substances of very high concern (SVHCs) under REACH—justification, risk-benefit analysis, and specific labeling are required [44].

Bisphenol A (BPA) is listed as an SVHC due to its established endocrine-disrupting potential. Although the BPA content in orthodontic retainers typically remains well below this threshold, MDR provisions have prompted manufacturers to reformulate materials to avoid BPA altogether, thereby reducing regulatory burden and potential market resistance.

**Material** **Standards and CE Marking**

Dental materials used in retainers must also comply with material-specific standards. ISO 20795-2:2013 (“Dentistry—Base polymers—Part 2: Orthodontic base polymers”) is the principal specification adopted in the EU (EN ISO 20795-2) for acrylics and polymeric components of orthodontic appliances [45]. This standard defines acceptable physical properties such as flexural strength and color stability and implicitly contributes to biocompatibility by limiting residual monomer content. For example, the permitted concentration of unreacted methyl methacrylate (MMA) in denture base polymers is typically capped at or below 2%.

Manufacturers seeking CE marking must demonstrate conformity with ISO 20795-2 and ISO 10993 standards, particularly for appliances intended for extended intraoral use. These regulatory benchmarks serve to ensure conformity with both mechanical integrity and biological safety requirements.

**ISO 10993 Biocompatibility** [46]

Both the FDA and EU regulatory pathways require compliance with ISO 10993 for biocompatibility evaluation. For orthodontic appliances with long-term mucosal contact (>30 days), relevant test panels include cytotoxicity, subacute and chronic systemic toxicity, mucosal irritation, and—if warranted—genotoxicity. Additional testing is recommended if novel chemical entities are introduced or if known endocrine-disrupting activity is suspected.

While independent research has occasionally reported low-level cytotoxic or estrogenic responses to dental polymers, these findings remain below the thresholds defined in ISO protocols [46]. Compliance with ISO 10993 criteria is generally regarded as indicative of clinical safety within prevailing regulatory frameworks.

**Labeling** **and Product Information**

In the EU, if a device contains an SVHC above 0.1% by weight, this must be disclosed on the product label and technical documentation [47]. Instructions for Use (IFUs) and Safety Data Sheets (SDSs) are expected to communicate the presence or absence of compounds such as BPA or phthalates. Manufacturers increasingly emphasize ‘BPA-free’ or ‘phthalate-free’ status in product documentation and promotional materials. In the U.S., such declarations are often voluntary, except in the case of recognized allergens such as latex [43].

**Professional** **and Clinical Guidelines**

Professional associations have acknowledged the emerging concerns around polymer degradation products and their systemic effects. Recent peer-reviewed investigations stress the need for continued materials innovation—ranging from direct 3D-printed aligners to improved analytical monitoring of monomer release—to further limit patient exposure to potentially harmful substances [48,49].

Current international standards provide a robust framework for evaluating the safety of orthodontic retainer materials. To date, no routinely used orthodontic appliance has been subjected to regulatory prohibition by either U.S. or EU authorities, indicating that observed levels of chemical leaching and cytotoxicity are within tolerable limits. Nonetheless, the EU MDR threshold for SVHC disclosure (>0.1%) represents an important regulatory driver pushing the industry toward cleaner formulations.

Clinicians are advised to remain informed about material composition and to consider BPA-free or hypoallergenic alternatives when treating sensitive individuals. While the overall benefit-risk profile of retainers remains favorable, regulatory expectations and public scrutiny are evolving. Continuous improvement in material safety and compliance with updated standards are therefore essential.

## 7. One Health and Environmental Perspective

In recent years, the evaluation of orthodontic retainer materials has extended beyond individual biocompatibility to encompass environmental and public health dimensions. This broader view, aligned with the One Health framework, highlights the interdependence of human, animal, and ecosystem health. Key concerns include microplastic release, chemical leaching, and the environmental persistence of polymer-based appliances [50,51].

**Microplastic** **Release**

Polymeric retainers degrade under intraoral mechanical stress and chemical exposure, releasing micro- and nanoplastic particles (MNPs). Ceccarelli et al. (2024) confirmed MNP detachment from aligner surfaces after simulated one-week use [52], while Barile et al. [39] observed polymer fragment shedding from various aligner brands subjected to cyclic loading. Most particles ranged from tens to hundreds of micrometers, but nano-sized fragments (<1 μm) may cross epithelial barriers. Their presence in human placenta and the bloodstream in unrelated contexts supports this possibility [53].

Although acute health effects appear minimal, the potential for chronic low-dose exposure, especially in younger users, remains underexplored. As clear-aligner therapy continues to expand globally, peer-reviewed studies are beginning to quantify both the chemical emissions and the microplastic load generated by orthodontic polymers [49,51].

**Chemical** **Accumulation and Wildlife Impact**

Retainers also act as diffuse sources of bisphenols and other leachable additives. Although the release from a single device is low, frequent appliance replacement—weekly during active treatment, semiannually in retention—amplifies the environmental burden. In landfill conditions, residual monomers like BPA can persist and leach into soil or groundwater.

Even trace concentrations of BPA disrupt endocrine function in aquatic organisms, inducing feminization and developmental changes at parts-per-trillion levels. While orthodontic appliances contribute modestly to total BPA emissions, their chemical stability and disposal rate underscore their relevance as an environmental source. As noted by [53], such devices are excluded from recycling streams due to their mixed composition and biohazard status, limiting their integration into circular waste systems.

**One Health** **Framework and Preventive Design**

Elimination of hazardous constituents in orthodontic materials offers concurrent benefits for patient safety, occupational exposure, and environmental integrity. BPA- and phthalate-free formulations lower chemical exposure for users and technicians, while chemically stable polymers reduce contamination risks via landfill leachates or wastewater. These strategies support a model of sustainable orthodontics that emphasizes material efficiency, toxicity reduction, and end-of-life accountability [54,55].

Industry responses include design-for-disposal approaches. A 2022 UK pilot by Align Technology implemented post-use collection of aligners for energy recovery or downcycling—an early move toward embedding sustainability in practice.

**Emerging** **Eco-Friendly Materials**

Biopolymer-based alternatives to petroleum-derived plastics are under development. Cellulose acetate thermoformable matrices show partial biodegradability and compatibility with antimicrobial additives. In vitro tests of cinnamaldehyde-loaded cellulose aligners confirmed biofilm inhibition without cytotoxicity [56,57]. Composites with nanohydroxyapatite and quaternary ammonium compounds have also demonstrated remineralizing and antibacterial effects while maintaining cell viability [58].

Despite promise, these materials require validation regarding mechanical durability, long-term biocompatibility, and nanoparticle release during wear and disposal, as environmental fate data remain limited.

**Green** **Dentistry Practices**

Reducing the ecological impact of orthodontics involves both materials and workflow. Digital impressions replace disposable trays, while optimized 3D printing reduces resin waste. Long-lasting retainers made from PEEK or laminated polymers may reduce replacement frequency and resource use. Such strategies embody life-cycle assessment principles by addressing both clinical effectiveness and environmental resource utilization [55].

Balancing patient preferences (e.g., frequent replacement of thin retainers) with sustainability is under discussion. The recent literature urges integration of environmental indicators into clinical decision-making.

**Environmental** **Regulations and Policy Outlook**

Although no specific environmental regulations target orthodontic appliances, broader legislation may influence future materials. EU Regulation 2017/745 requires justification and labeling for devices containing >0.1% SVHCs, including BPA. ISO 10993 standards remain central to biocompatibility testing, but regulatory focus is expanding to encompass life-cycle impacts [44,46].

**Conclusion** 

The One Health framework situates orthodontic biomaterials within interlinked human and environmental systems. While current polymers meet intraoral safety standards, their long-term ecological persistence and chemical activity warrant scrutiny. Emerging materials offer safer, potentially more sustainable options, but require a balance between clinical utility and environmental responsibility.

Orthodontic material development is moving toward integration of performance with ecological safety. Realizing this shift will depend on coordinated efforts among researchers, manufacturers, and regulators to ensure innovations benefit both oral health and planetary systems.

## 8. Environmental Implications

Beyond the patient-level effects, removable retainers and clear aligners raise broader environmental health questions. These devices are polymers that eventually become plastic waste. Peer-reviewed life-cycle analyses now quantify that burden and place aligner plastics within broader discussions of medical-polymer waste [59].

Microplastics and Nanoplastics Release: Orthodontic polymer-based appliances are continuously exposed to mechanical loads (e.g., mastication, parafunction) and intraoral chemical challenges, including enzymatic activity and thermal fluctuation. Over time, this can cause the plastic surface to degrade and microscopic fragments to shed. Recent studies have confirmed that aligners do release microplastic particles during use. Quinzi et al. found that after 7 days of simulated use, various aligner brands released particles in the 5–20 µm size range (microplastics) detectable by spectroscopic analysis. The quantity of released particles varied by brand, with certain materials exhibiting greater degradation propensity; one formulation released markedly more microparticles than Invisalign, which showed the lowest emission [52]. The released microparticles may undergo ingestion or become incorporated into oral biofilms. The in vivo significance is not fully known, but the ingestion of microplastics, in general, has been linked to inflammation and potential uptake into tissues.

Plastic Waste and Disposal: Treatment with clear aligner systems may involve sequential use of 20–30 appliance sets per patient (upper and lower) over a treatment. Each aligner weighs a few grams (one source says about 4.3 g per pair) [53]. That is ~100–130 g of plastic per patient per treatment. If the global clear aligner market treated in the order of, say, 1 million patients per year (a hypothetical figure), that is over 100 metric tons of plastic waste generated per year from aligners alone. Even retention phase involves periodic replacement of Essix retainers (which might be replaced every 6–12 months if they wear out). Currently, most discarded aligners and retainers end up in landfills or regular garbage [53]. Recycling pathways for these devices are generally unavailable, in part due to their classification as biohazardous waste (having been in the mouth) and are multi-material (some have embedded metal or composites). Under landfill conditions, the constituent polymers exhibit long-term environmental persistence and limited biodegradability.

Public Health and Ecosystem Impact: The issue of microplastics is a public health concern, as they have been found in water supplies and even human tissue. However, aligners are a relatively small fraction of total plastic pollution (far overshadowed by packaging, bottles, synthetic clothing fibers, etc.). Nonetheless, sustainability considerations are gaining prominence within contemporary orthodontic practice. Macrì et al. [53] introduced the “4Rs” strategy—Reduce, Reuse, Recycle, Rethink—as a conceptual framework to mitigate the environmental footprint of aligner therapy. Proposed interventions include optimizing treatment protocols to minimize material consumption, exploring repurposing options for used trays, establishing recycling systems despite logistical challenges, and advancing the transition toward biodegradable polymer alternatives. From a regulatory perspective, if BPA or other SVHCs are present in the devices, their environmental release is also concerning. For instance, the EU’s restriction on BPA in consumer goods (like the ban on baby bottles and limits on food contact materials) is partially due to environmental persistence. Orthodontic devices have not been targeted specifically, but manufacturers likely proactively removed BPA to preempt potential future regulatory or legal challenges.

## 9. Conclusions and Future Directions

In recent years, increasing attention has been directed toward the biocompatibility of orthodontic retainer materials, reflecting evolving safety standards and scientific awareness. The evidence reviewed indicates that removable retainers made of PMMA-based acrylic or PETG/TPU thermoplastics are generally safe, with only mild cytotoxic and estrogenic effects observed. Neither Hawley nor Essix-type appliances show any severe adverse biological impact on patients; these appliances have been used extensively in clinical practice without reports of significant adverse effects. Nevertheless, the detection of oxidative stress biomarkers, subcellular alterations, and trace bisphenol release indicates that these materials exhibit limited but measurable biological reactivity. Although these materials exhibit generally favorable biocompatibility, measurable interactions with the oral environment indicate opportunities for further refinement in their composition and safety profile.

**Evidence-Based** **Clinical Recommendations**

- Both major retainer types (Hawley and Essix) are associated with slight cytotoxicity in vitro and minor biomarker changes in vivo, but no overt pathology. Patients can be reassured of their overall safety, while practitioners remain vigilant for rare sensitivities or allergies.
- Residual monomer release is the main issue with acrylic Hawley retainers. Using heat-cured acrylic and allowing the appliance to soak in water (or even in saliva in the mouth for a while before full-time wear) can reduce initial exposure. If a patient complains of a strong acrylic taste or burning, the retainer can be soaked longer or remade with better curing.
- BPA and xenoestrogens can leach from some clear thermoplastics, especially within the first day of use. To mitigate this, clinicians should consider rinsing/soaking new clear retainers before delivery. Additionally, selecting products that are BPA-free (and have data to back it up) is wise. If a particular thermoplastic has known higher leach rates, alternatives should be used, particularly for young patients or those desiring pregnancy, etc., as a precautionary measure, particularly in susceptible populations.
- Monitoring and maintenance: During retainer check visits, inspect the mucosa for any signs of chronic irritation. In long-term wearers, ensure that any inflammatory issues are addressed (sometimes simply polishing the edges of an Essix or adjusting a Hawley can remove physical irritation that might exacerbate cellular stress).
- Patient communication: Educate patients on the importance of cleaning their retainers daily—not just for hygiene, but also because plaque and calculus on the appliance can cause gum inflammation and could interact with any leached substances. A clean appliance is less likely to cause any tissue response beyond what the material itself does.
- Consider material alternatives for sensitive patients. For example, if someone has a history of acrylic allergy (to nail acrylics, etc.), a polypropylene-based Essix retainer may be preferable in such cases (which has virtually no leachable monomer) instead of a Hawley. If a patient is concerned about plastic, a fixed retainer could be an option to eliminate a removable plastic device.

**Forward-Looking** **Considerations and Research Directions**

- Continued surveillance of novel materials is warranted, particularly those incorporating antimicrobial or bioactive agents. Importantly, any new additive requires thorough toxicological validation to preclude the introduction of unintended biocompatibility concerns.
- The proposal for biodegradable or recyclable orthodontic materials is a promising development consistent with principles of environmental sustainability. However, it is important to note that current biodegradable polymers often lack the mechanical durability and transparency required for long-term orthodontic applications. Thus, while environmentally desirable, biodegradable retainers remain a future goal that must be balanced with clinical practicality, patient safety, and cost-effectiveness.
- Future in vivo studies should explore long-term wear effects, including oxidative stress persistence and any systemic biomarkers.
- Mechanistic studies are needed to clarify the pathways through which specific additives exert cytotoxic or estrogenic effects.
- Regulatory evolution may necessitate lower tolerances for BPA and other leachables, urging manufacturers toward reformulated materials.
- From a public health perspective, the orthodontic community should aim to minimize even minimal risks, especially in pediatric and adolescent populations.

Future research should prioritize the development of standardized testing protocols for biocompatibility and endocrine-disrupting properties to enable consistent and comparable evaluation across orthodontic materials. In conclusion, Hawley and Essix retainers remain safe and effective appliances as supported by current research, but ongoing improvements in material science and awareness of biocompatibility will continue to enhance their safety profile. Adherence to evidence-based practices and informed material selection can enhance both the clinical efficacy and biocompatibility of retention therapy, while also aligning with environmental sustainability objectives. Progress in polymer science is expected to produce materials with enhanced biocompatibility, potentially mitigating present concerns regarding cytotoxic and endocrine-disrupting properties in orthodontic appliances.

## Figures and Tables

**Table 1:** Composition, leachable components, biocompatibility profile, and relative cytotoxicity of removable orthodontic retainer materials.

Retainer Material	Composition and Key Components	Potential Leachables	Notable Biocompatibility Considerations	Relative Cytotoxicity
Hawley retainer (PMMA base + wire)	PMMA acrylic (polymerized methyl methacrylate) baseplate; stainless steel wire clasps. Typically autopolymerized (cold-cure) or heat-cured.	Residual methyl methacrylate (MMA) monomer; other additives (e.g., peroxide initiator residues, pigments). No BPA inherently in PMMA.	Residual monomer can cause cytotoxic and irritant effects on oral cells [8]. Chemical-cure acrylics leave more MMA (greater cell toxicity) than heat-cure [2]. Rare allergic reactions to acrylic reported. Generally good long-term biocompatibility once fully cured.	Moderate
Essix retainer (PET-G thermoplastic)	Thermoformed PET-G (polyethylene terephthalate glycol) sheet. Petroleum-based transparent polymer, often ~1 mm thick.	Trace ethylene glycol or terephthalate oligomers; any added UV stabilizers or colorants. Base polymer is BPA-free, but some formulations may include additives derived from BPA for clarity [12].	Considered inert and stable; very low cytotoxicity in vitro. However, one clinical study found measurable BPA in saliva of patients with PET-G retainers [2] (likely from additives or contamination). Generally low incidence of mucosal irritation.	Low
Essix retainer (polypropylene or polyethylene)	Some vacuum-formed retainers use polypropylene or polyethylene blends (softer, flexible thermoplastics).	Minimal (polyolefins have very low leachables). No BPA or phthalates typically needed.	Polypropylene retainers have shown minimal cytotoxicity. However, lower stiffness can allow more bacterial plaque adherence. Biocompatibility is high; issues primarily mechanical (wear/tear) rather than chemical.	Very Low
Clear aligner-type (polyurethane, e.g., Invisalign)	Multilayer aliphatic or semi-aromatic thermoplastic polyurethane (TPU). Often proprietary blends; e.g., Invisalign’s SmartTrack is a multi-layer polyurethane.	Oligomers or degradation products of urethane (e.g., 1,4-butanediol) under extreme conditions. No BPA or phthalate plasticizers by design [13].	TPU aligners exhibit slight cytotoxicity to cells in vitro (comparable to PETG).	Low
3D-printed retainer (acrylate resin)	Photopolymerized resin (e.g., urethane dimethacrylate-based). Custom printed to fit teeth, then post-cured. | Unpolymerized methacrylate monomers (if curing incomplete); photoinitiator chemicals; possible bisphenol-A derivatives if present in resin	If properly cured and washed, can be safe; however, studies note variability. Some printed dental resins leach compounds causing higher cytotoxic and even estrogenic effects than thermoplastics [14].	Thorough post-processing (wash, UV cure) is critical to reduce toxicity. Not yet widely used pending further biocompatibility validation.	Moderate to High *

* Depending on curing process and residual monomer levels.

**Table 4:** Overview of removable orthodontic retainer types, associated chemical components, and reported biological risks (cytotoxicity, endocrine activity), based on in vitro and clinical evidence.

Retainer Type	Material	Common Monomers/Additives	Potential Risks
Hawley Retainer	PMMA + stainless steel wire	Methyl methacrylate (MMA), residual monomers	Cytotoxicity, allergic reactions (e.g., contact stomatitis), MMA leaching
Essix (C+) Retainer	PVC-based thermoplastic	Phthalates, residual vinyl chloride	Endocrine disruption, possible release of plasticizers
Essix ACE Retainer	Copolyester (PETG-based)	BPA, PETG oligomers	Low-level BPA release, minor cytotoxicity
Modern orthodontic thermoplastics (Duran^®^, Essix ACE^®^, Zendura^®^ FLX, etc.)	Polyurethane	BPA, BPS	Potential estrogenic activity (in vitro), low cytotoxicity
3D-printed retainer (acrylate resin)	Multilayer polyurethane (proprietary)	BPA analogs (BPS, BPF?)	Unclear—proprietary composition, risk depends on aging, wear

## Data Availability

The datasets used and/or analyzed during the current study are available from the corresponding author upon reasonable request.
